# Effect of donor-recipient relatedness on the plasmid conjugation frequency: a meta-analysis

**DOI:** 10.1186/s12866-020-01825-4

**Published:** 2020-05-26

**Authors:** Jesse B. Alderliesten, Sarah J. N. Duxbury, Mark P. Zwart, J. Arjan G. M. de Visser, Arjan Stegeman, Egil A. J. Fischer

**Affiliations:** 1 Department of Population Health Sciences, Faculty of Veterinary Medicine, Utrecht University, Utrecht, The Netherlands; 2 Laboratory of Genetics, Wageningen University, Wageningen, The Netherlands; 3 Department of Microbial Ecology, The Netherlands Institute of Ecology (NIOO-KNAW), Wageningen, The Netherlands

**Keywords:** Plasmid, Horizontal gene transfer, Antibiotic resistance, *Escherichia coli*

## Abstract

**Background:**

Conjugation plays a major role in the transmission of plasmids encoding antibiotic resistance genes in both clinical and general settings. The conjugation efficiency is influenced by many biotic and abiotic factors, one of which is the taxonomic relatedness between donor and recipient bacteria. A comprehensive overview of the influence of donor-recipient relatedness on conjugation is still lacking, but such an overview is important to quantitatively assess the risk of plasmid transfer and the effect of interventions which limit the spread of antibiotic resistance, and to obtain parameter values for conjugation in mathematical models. Therefore, we performed a meta-analysis on reported conjugation frequencies from *Escherichia coli* donors to various recipient species.

**Results:**

Thirty-two studies reporting 313 conjugation frequencies for liquid broth matings and 270 conjugation frequencies for filter matings were included in our meta-analysis. The reported conjugation frequencies varied over 11 orders of magnitude. Decreasing taxonomic relatedness between donor and recipient bacteria, when adjusted for confounding factors, was associated with a lower conjugation frequency in liquid matings. The mean conjugation frequency for bacteria of the same order, the same class, and other classes was 10, 20, and 789 times lower than the mean conjugation frequency within the same species, respectively. This association between relatedness and conjugation frequency was not found for filter matings. The conjugation frequency was furthermore found to be influenced by temperature in both types of mating experiments, and in addition by plasmid incompatibility group in liquid matings, and by recipient origin and mating time in filter matings.

**Conclusions:**

In our meta-analysis, taxonomic relatedness is limiting conjugation in liquid matings, but not in filter matings, suggesting that taxonomic relatedness is not a limiting factor for conjugation in environments where bacteria are fixed in space.

## Background

Antibiotic resistance (ABR) in bacteria is recognised world-wide as an important threat to human and animal health [1–3]. To address this threat, a better understanding of the key factors that determine the spread of ABR between bacteria is needed. A key factor in the spread of ABR is the transmission of plasmids that encode ABR genes [4], which is the focus of this review.

Transmission of ABR genes between bacteria can occur through transformation, transduction, or conjugation. Transformation and transduction have been implicated in the spread of ABR in clinical settings, but their importance is not yet clear [5–7]. In contrast, the major role of conjugation in the spread of ABR genes in both clinical and general settings is evident [5–7].

Conjugation efficiency is most commonly quantified by the ratio of the number of transconjugants (i.e., recipient cells that have received a plasmid from a donor cell) at the end of the experiment to the number of donors or recipients at the beginning of the experiment. We will call this the conjugation frequency, but we note that in the literature it has been called conjugation rate as well. We will use the term conjugation rate for the speed of the conjugation process, with units mL cell^− 1^ h^− 1^.

The conjugation frequency is determined in mating experiments, where donors and differently-marked recipient bacteria are separately cultured, then mixed in liquid [8–12], on agar medium [12], on filters [12–14], on touch surfaces [15], or in animals [9, 16, 17] and allowed to conjugate for a given period. Then the different population densities are determined by selective plating, and the conjugation frequency is quantified by the ratio of the number of transconjugants to the initial number of donors or recipients. This can be done at multiple time points during the experiment to obtain a time series, or at the end of the experiment to obtain a single estimate of the conjugation frequency.

Mating experiments have shown that the conjugation frequency is affected by various biotic and abiotic factors, such as growth phase, cell density, donor-to-recipient ratio, carbon and metal concentrations, temperature, pH, and mating time [11, 18–21]. The used donor and recipient species, the plasmid, and the use of liquid matings, filter matings, or matings in live animals as experimental method also significantly influence the conjugation frequency [22].

The conjugation efficiency of plasmids is limited by the various steps involved [23]. The donor has to meet a recipient bacterium, form a conjugative pilus, and attach to the surface of the recipient bacterium. The probability of mating-pair formation is influenced by the density of donors and recipients, their motility, and the structure of the environment (i.e., liquid versus solid, or structure of the filter [24]). Once a mating pair has been successfully formed, a copy of the plasmid has to be transferred to the recipient, and the pilus should remain intact until this process is finished. Plasmids carrying genes that code for all the machinery needed to form a mating pair and transfer the plasmid to the recipient are called self-transmissible plasmids, whereas plasmids that require the help of transfer machinery encoded on other plasmids in the donor bacterium to achieve this are called mobilisable plasmids. Once inside the recipient, the plasmid should escape degradation by restriction endonucleases of the recipient which recognise restriction sites on the plasmid, and host factors should be able to ensure plasmid replication and equal distribution of the plasmid copies among the two daughter cells during cell division [23].

The conjugation efficiency can also be affected by plasmids that are already present in the recipient bacterium. They can stabilise mating pairs and increase the conjugation efficiency [25], or decrease mating-pair formation and make it more difficult for other related plasmids to enter the recipient [23]. Plasmids in the recipient can inhibit stable maintenance of other plasmids if they use the same replication-control mechanism [26]. Based on the different replication-control mechanisms, 28 different incompatibility (Inc) groups are recognised for plasmids in *Enterobacteriaceae* [27]. The presence of genes coding for replication-control mechanisms correlates with the presence of genes needed for conjugation [28], and therefore may correlate with differences in conjugation efficiency.

A potentially fundamental and generic determinant of conjugation efficiency is the taxonomic relatedness between donor and recipient bacteria. On evolutionary timescales, plasmid genes are more frequently shared within than between taxonomic classes, and even more frequently between lower taxa [29]. Recently shared mobile resistance genes are also more frequently shared within than between taxa, from the species level up to the phylum level [30]. This effect of taxonomic relatedness is apparent when comparing conjugation frequencies within versus between genera [22], and also when comparing conjugation frequencies at the intraspecies level between transconjugants and recipients from which they were derived versus donors and recipients [8]. The latter could be caused by de-repression of plasmid genes in transconjugants, leading to a temporarily higher conjugation rate in transconjugants [8]. It could also be caused by the shared genetic background of the transconjugants and recipients, as opposed to the different genetic backgrounds of the donors and recipients. This could be important, since small genetic differences at the strain level determine the restriction status of the recipient, which affects intraspecies conjugation rates more than the genetic distance between them [31].

Conjugation efficiency is clearly influenced by many factors, one of which is the taxonomic relatedness between donor and recipient bacteria. Understanding the role of relatedness is important in order to determine the potential of ABR plasmids to spread between species. However, a comprehensive overview of the influence of taxonomic relatedness between donor and recipient bacteria on the conjugation frequency is still lacking. Such an overview is important to quantitatively assess the risk of plasmid transfer [22] and the effect of interventions which limit the spread of ABR [32, 33], and to obtain parameter values for conjugation in mathematical models [33, 34]. We performed a meta-analysis on reported conjugation frequencies from *Escherichia coli* (*E. coli*) donors to various recipient species, incorporating taxonomic distances from the intraspecies up to the phylum level, and taking into account differences in biotic and abiotic factors between studies.

## Results

### Identification of relevant studies

**Fig. 1:**
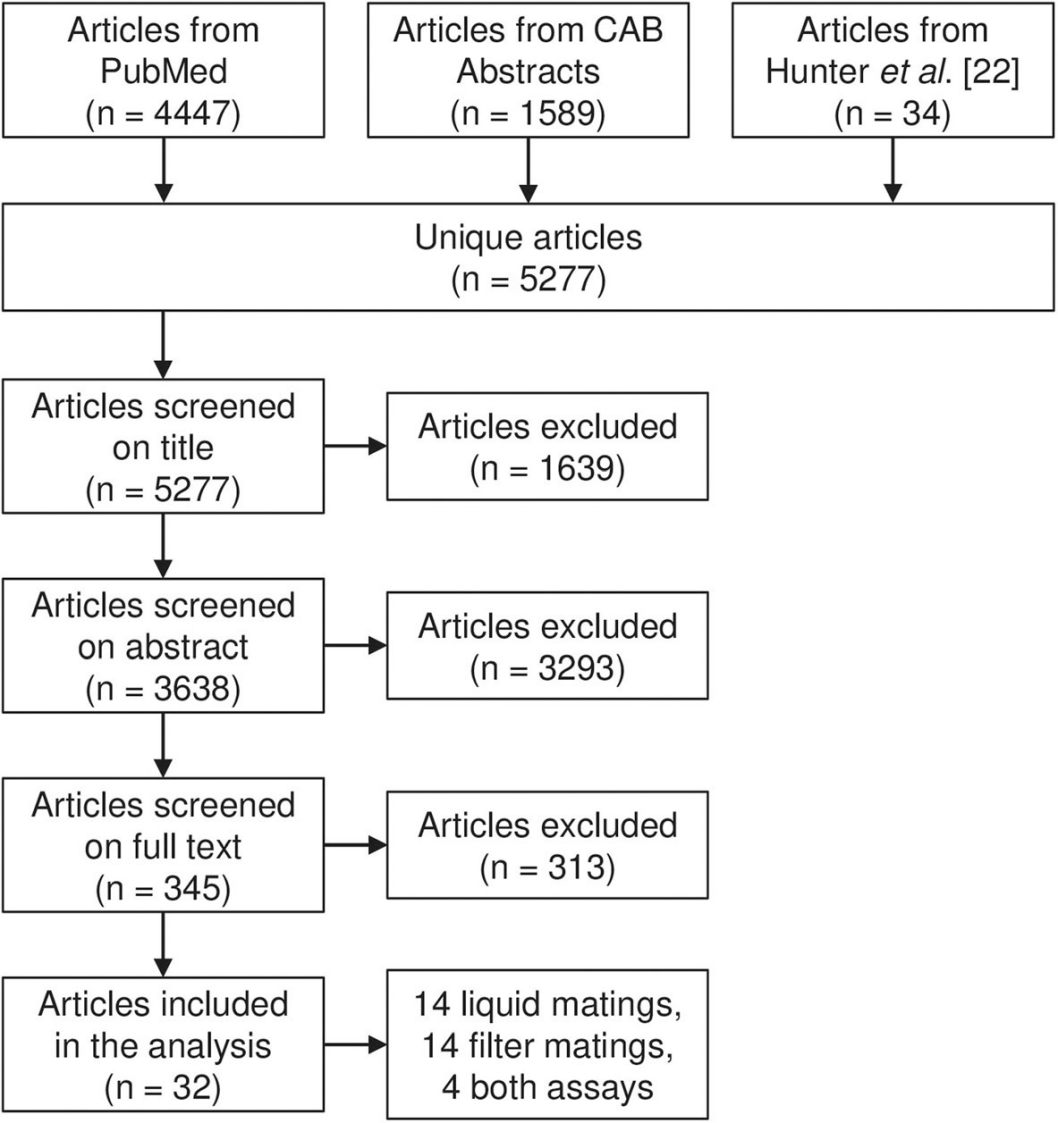
Flowchart depicting the search results and the selection of studies. N: number of studies

Our selection for studies which mentioned more than one recipient species in the abstract, used liquid broth matings or filter matings with *E. coli* donors containing a self-transmissible plasmid yielded 32 studies (Fig. 1) reporting 313 conjugation frequencies for liquid broth matings [9, 35–51] and 270 conjugation frequencies for filter matings [38, 44, 45, 50, 52–65].

### Liquid broth matings

Across all liquid broth matings, the conjugation frequencies varied over nine orders of magnitude (1.0 · 10^− 9^ – 1.3; Additional file 1). The conjugation frequency was below the detection limit in 80 out of 313 cases (25.6%).

The final statistical model to test the effect of donor-recipient relatedness on the conjugation frequency in liquid broth matings contained the following factors: taxonomic relatedness to *E. coli*, donor origin, plasmid Inc group, temperature, and agitation of the medium (Table 1). The mean conjugation frequency was lower for more distantly related recipients, albeit in a non-monotonic manner. The mean conjugation frequency for bacteria of the same family, the same order, the same class, and other classes was 0.37, 10, 20, and 789 times lower than the mean conjugation frequency within the same species, respectively (Table 1). Other factors that significantly influenced the conjugation frequency were plasmid Inc group and temperature. Plasmids from Inc groups A/C had a 85 times lower conjugation frequency than IncF plasmids, and plasmids from unreported Inc groups had a 93 times lower conjugation frequency than IncF plasmids. The conjugation frequency was 14 times higher at 20–30 °C than at 35–37 °C (Table 1).

**Table 1:** Parameter estimates for the final multivariable linear mixed regression model for liquid broth matings

	Included data points		Log_10_(T / D)		
	n	%	Mean	95% CI	
**Reference**					
	313	100	−4.97	−6.73	−3.22
**Relatedness to*E. coli*donor**					
Same species	184	58.8	Reference		
Same family	47	15.0	0.43	−0.25	1.12
Same order	43	13.7	−1.00	− 1.93	−0.08
Same class	23	7.3	−1.30	−3.53	0.93
Other class	16	5.1	−2.90	−5.19	−0.60
**Donor origin**					
Laboratory strain	222	70.9	Reference		
Chicken	71	22.7	−1.70	−5.15	1.76
Human	16	5.1	0.06	−2.78	2.90
Wastewater	4	1.3	2.42	−1.86	6.69
**Plasmid Inc group**					
F	80	25.6	Reference		
A/C	7	2.2	−1.93	−3.33	−0.54
H	60	19.2	−1.38	−4.91	2.16
I	1	0.3	−0.38	−3.77	3.01
L/M	8	2.6	−0.06	−1.38	1.27
P	39	12.5	1.90	−0.71	4.51
T	6	1.9	−2.22	−6.37	1.94
V	3	1.0	−3.56	−7.90	0.78
X	2	0.6	2.62	−1.52	6.75
NR	107	34.2	−1.97	−2.94	− 1.00
**Temperature (°C)**					
5	4	1.3	0.86	−0.95	2.68
20–30	171	54.6	1.15	0.55	1.75
35–37	119	38.0	Reference		
42–47	12	3.8	−0.97	−2.04	0.11
NR	7	2.2	−1.44	−5.12	2.25
**Agitation of the medium**					
Shaken	143	45.7	Reference		
Static	70	22.4	−0.70	−2.35	0.94
NR	100	31.9	2.13	−0.89	5.14

*CI* confidence interval; *D* initial number of donors; *Inc* incompatibility; *n* number of data points; *NR* not reported; *T* number of transconjugants. The value given as ‘Reference’ denotes the mean log_10_(T/D) if all variables are equal to their reference category, i.e., if the recipient is an *E. coli* bacterium, the donor is an *E. coli* laboratory strain carrying an IncF plasmid, and the experiment is performed at 35–37 °C in shaken medium. The values for the other categories list the differences from that overall mean

### Filter matings

Across all filter matings, the conjugation frequencies varied over 11 orders of magnitude (4.5 · 10^− 11^ – 2.1; Additional file 1). The conjugation frequency was below the detection limit in 97 out of 270 cases (35.9%).

The final statistical model to test the effect of donor-recipient relatedness on the conjugation frequency in filter matings contained the following factors: taxonomic relatedness to *E. coli*, recipient origin, donor-to-recipient ratio, temperature, mating time, and pore size of the filter (Table 2). The mean conjugation frequency for bacteria of the same family as *E. coli* was 41 times higher than the mean conjugation frequency within the same species, but relatedness did not significantly influence the conjugation frequency for the other taxonomic groups. Other factors that significantly influenced the conjugation frequency were recipient origin, temperature, and mating time. Recipients obtained from dairy cattle, water, and food had a 143, 1.25 · 10^6^, and 197 times lower conjugation frequency than laboratory strains, respectively. The conjugation frequency was 3036 times higher at 5 °C than at 35–37 °C. The conjugation frequency was 4239 times lower for experiments with a mating time of 16–24 h than for experiments with a mating time of less than 4 h (Table 2).

**Table 2:** Parameter estimates for the final multivariable linear mixed regression model for filter matings

	Included data points		Log_10_(T / D)		
	n	%	Mean	95% CI	
**Reference**					
	270	100	−5.38	−9.37	−1.40
**Relatedness to*E. coli*donor**					
Same species	130	48.1	Reference		
Same family	88	32.6	1.61	0.74	2.47
Same class	22	8.1	0.59	−1.03	2.22
Other class	30	11.1	0.64	−5.37	6.65
**Recipient origin**					
Laboratory strain	116	43.0	Reference		
Human	57	21.1	−0.86	−1.80	0.08
Dairy cattle	44	16.3	−2.16	−3.37	−0.94
Plant	16	5.9	−2.67	−6.75	1.41
Water	10	3.7	−6.10	−7.89	−4.31
Food	5	1.9	−2.29	−4.20	−0.39
Acidic drainage of a hot-spring	2	0.7	−1.37	−8.50	5.77
NR	20	7.4	−2.26	−4.56	0.03
**Donor-to-recipient ratio**					
0.05–0.125	80	29.6	−2.59	−6.48	1.30
0.25–0.50	54	20.0	2.42	−0.83	5.68
1.00	124	45.9	Reference		
3.00	10	3.7	−1.94	−8.31	4.44
NR	2	0.7	1.48	−3.50	6.46
**Temperature (°C)**					
5	3	1.1	3.48	1.44	5.52
20–30	49	18.1	−0.15	−1.90	1.60
35–37	190	70.4	Reference		
42–47	6	2.2	−0.03	−1.48	1.41
NR	22	8.1	1.06	−1.67	3.80
**Mating time (h)**					
≤ 4	102	37.8	Reference		
4.01–8.0	17	6.3	−1.80	−5.97	2.38
16–24	114	42.2	−3.63	−6.94	−0.31
30	27	10.0	−3.21	−7.70	1.29
48–72	10	3.7	−2.99	−10.78	4.81
**Filter pore size (μm)**					
0.20–0.22	17	6.3	Reference		
0.40–0.45	229	84.8	3.51	−1.35	8.38
NR	24	8.9	2.61	−2.59	7.82

*CI* confidence interval; *D* initial number of donors; *n*: number of data points; *NR* not reported; *T* number of transconjugants. The value given as ‘Reference’ denotes the mean log_10_(T/D) if all variables are equal to their reference category, i.e., if the recipient is an *E. coli* laboratory strain, and the experiment is performed with a donor-to-recipient ratio of 1.00, a temperature of 35–37 °C and less than 4 h mating time on a filter with a 0.20–0.22 μm pore size. The values for the other categories list the differences from that overall mean

## Discussion

Decreasing taxonomic relatedness between donor and recipient bacteria is associated with a lower conjugation frequency in liquid matings, but not in filter matings, when adjusted for confounding factors (Table 1; Table 2). This distinction between liquid and filter matings regarding the influence of relatedness can be explained by the different conditions in these two assays with respect to mating-pair formation. The efficiency of mating-pair formation is influenced by local cell density, as well as by the lipopolysaccharides and outer-membrane proteins at the cell surface of the recipients. The type of conjugative pili (thin flexible, thick flexible, or rigid) might also play an important role [66], but in the reviewed studies pilus type was not determined and pilus type cannot be inferred otherwise with confidence [67, 68].

The efficiency of intraspecies mating-pair formation in liquid broth can be decreased by mutations in the lipopolysaccharide pathway, which decrease recipient ability in *E. coli* [69]. This could explain why less-related bacteria are less-efficient at mating-pair formation in liquid matings, resulting in a lower conjugation frequency. This effect, however, is absent in solid plate matings [69]. Similarly, differences in the lipopolysaccharide structure of *Salmonella typhimurium* strains can affect their mating-pair formation abilities. Normal *Salmonella typhimurium* strains, which are not able to form stable mating pairs in liquid, do not conjugate efficiently in liquid matings, but these strains do conjugate efficiently in filter matings. In contrast, mutant *Salmonella typhimurium* strains lacking lipopolysaccharide side chains conjugate efficiently in both liquid broth and filter matings [50]. This also points to mating-pair formation as a limiting step in conjugation in liquid matings. These differences in mating-pair formation efficiency between different strains of the same species in liquid mating can also lead to competition between donors, related recipients and less-related recipients for mating-pair formation with a donor. In liquid matings, this competition will be present over the whole period of the experiment because of the constant mixing of the populations.

In filter matings, the bacteria are fixed in space such that conjugation is limited to neighbouring cells. Under these conditions, mating-pair formation does not play an important role in limiting conjugation, because conjugation to related recipients will be efficient at first and will then saturate when all neighbouring recipients have received the plasmid. Conjugation to less-related recipients might be less efficient, but because the bacteria are fixed in space, conjugation will continue for a longer time, and cease when all neighbouring recipients have received the plasmid. Competition for mating-pair formation will be less important here as well, because the mating pairs are fixed in space. As a result, the difference in conjugation frequency to more-related versus less-related recipients will decrease over time, and at later time points relatedness will appear not to influence the conjugation frequency.

The conjugation frequency is slightly higher in filter matings than in liquid matings (Table 1; Table 2). In filter matings, the conjugation frequency reflects the intrinsic conjugation rate, i.e., the conjugation rate from one cell to another given that these cells form a mating pair [12, 70]. In liquid matings, the conjugation frequency reflects the product of the intrinsic conjugation rate upon mating-pair formation and the rate of mating-pair formation, and is therefore lower than the intrinsic conjugation rate itself [70]. Both assays thus have an important interpretation, where liquid matings can be used to quantify conjugation rates and assess the effect of mating-pair formation in randomly mixing planktonic populations, and filter matings can be used to determine the ability of conjugation and to quantify the intrinsic conjugation rate, when the number of neighbouring recipients is limited.

In our review, conjugation was quantified by the ratio of the number of transconjugants to the initial number of donors. In order to better understand the role of conjugation in the spread of antibiotic-resistance plasmids, the way conjugation is quantified needs attention. The ratio of the number of transconjugants to the initial number of donors or recipients is a straightforward way to compare the ability of different strains to conjugate, but only when these assays are done under the same conditions. The same conditions are needed between experiments because the conjugation frequency ignores essential population dynamics that are influenced by experimental conditions such as initial densities of donors and recipients, donor-to-recipient ratio, nutrient concentration, and mating time [11, 71].

Levin et al. proposed a way to estimate the conjugation rate as a measure which is not sensitive to those experimental conditions, and Simonsen et al. elaborated on this by proposing a different method to measure it [11, 71]. This conjugation rate parameter is estimated from the growth rate and initial density of the total population and the densities of donors, recipients, and transconjugants at the end of the experiment, and is expressed in the units mL cell^− 1^ h^− 1^ [11]. It assumes random mixing of the bacteria, a resource-dependent growth rate which is the same for all strains, and a resource-dependent conjugation rate which is the same for donor and transconjugant strains [11, 71]. We could not calculate this parameter for any of the included studies, because growth rates of the bacteria and their densities at the end of the experiments were not reported. Therefore, we adjusted for differences in experimental conditions by including some of the experimental conditions as fixed effects in our statistical model. Some of the unexplained variance may be explained by factors such as the growth phases and initial densities of the bacteria and the pH of the medium, which could not be included in the model-selection process because they were frequently not reported. The random study effect also incorporates some of these potential effects of excluded variables on the conjugation frequency. The inclusion of this random study effect improved the model fit, showing that experimental conditions influenced the conjugation frequency.

The intestines are considered an important hotspot for the transmission of resistance plasmids with consequences for public and veterinary health [72, 73]. Conjugation of plasmids carrying extended-spectrum beta-lactamase genes is more efficient in the intestines than in liquid matings [8, 16], and in vivo transmission of plasmids in the intestines occurs in a way resembling a fixed spatial location such as in a biofilm [74]. Together with our finding that conjugation in filter matings is not affected by donor-recipient relatedness, this could suggest that distantly related bacteria which live together in the intestinal mucus exchange resistance plasmids through conjugation over large taxonomic distances. Maintenance in the transconjugant population is further influenced by factors such as fitness effects of the plasmid, adaptive evolution [75], segregational loss, and the presence of addiction systems [76]. Transconjugants from a distantly related recipient maintained the plasmid for 50 generations in absence of antibiotics [60], suggesting the possibility of long-term maintenance.

## Conclusions

Our results show that taxonomic relatedness is limiting conjugation in liquid matings, but not in filter matings, suggesting that relatedness is not a limiting factor for conjugation in environments where bacteria are fixed in space and conjugation is limited to mating between neighbouring bacteria.

## Methods

### Scope

We included studies in which *E. coli*, a medically and veterinary relevant species in which many resistance plasmids have been described [26, 77–79], was used as a donor. Furthermore, we restricted our search to liquid broth matings and filter matings, to circumvent the large heterogeneity regarding hosts and sampling methods encountered in in vivo studies. The data from the liquid broth matings and filter matings was analysed separately, because these assays represent different experimental systems with fundamental differences in mating opportunities.

### Search strategy

PubMed [80] and the CAB Abstracts database [81] were searched to identify relevant studies from the biomedical and veterinary field. Search terms to select articles giving quantitative data on conjugation of plasmids were combined with the AND-operator in a search which was restricted to the title and abstract: (1) dynamic*[tiab] OR efficienc*[tiab] OR rate*[tiab] OR kinetic*[tiab] OR frequenc*[tiab] OR model*[tiab] OR quantitat*[tiab] OR quantification*[tiab]; (2) (conjuga*[tiab] OR filter mating*[tiab] OR HGT[tiab]) OR ((horizontal*[tiab] OR lateral*[tiab] OR interspecific[tiab] OR interspecies[tiab]) AND(transfer*[tiab] OR spread*[tiab] OR transmiss*[tiab])) and (3) ((plasmid*[tiab]) OR ((resistan*[tiab] AND gene*[tiab]) OR (conjugative[tiab] AND transposon*[tiab]))) in PubMed and (1) (dynamic* or efficienc* or rate* or kinetic* or frequenc* or model* or quantitat* or quantification*).ab,ti.; (conjuga* or filter mating* or HGT or ((horizontal* or lateral* or interspecific or interspecies) and (transfer* or spread* or transmiss*))).ab,ti.; and (3) (plasmid* or ((resistan* and gene*) or (conjugative and transposon*))).ab,ti. in CAB Abstracts. The last search was performed on 18 September 2019. The 34 studies included in the review by Hunter et al., who focused on conjugation in the intestines and on intestinal bacteria not restricted to *E. coli* as a donor [22], and a study by Saliu et al. [49], were added to the search results as well.

### Study selection

The studies were imported into Covidence systematic review software [82]. Duplicate entries were removed. The remaining studies (*n* = 5277) were first screened for eligibility based on their title (Fig. 1). Studies were excluded if the title implied they did not deal with bacterial conjugation, or conjugation events from the distant evolutionary past were inferred by comparing genome sequences of bacteria, instead of measuring the conjugation efficiency in a laboratory assay.

The remaining studies (*n* = 3638) were then screened for eligibility based on their abstract. Studies that mentioned only one recipient species in the abstract were excluded, to select for studies in which the effect of relatedness on the conjugation frequency could also be assessed within the study. This approach allows us to make the comparison between studies less biased by methodological differences that will affect comparisons of conjugation frequencies. Studies were also excluded if the donor or recipient contained other plasmids apart from the one under study, or if the plasmid was not self-transmissible. The effects of surface-exclusion, plasmid incompatibility, and mobilisation on the conjugation frequency were thereby excluded as we wanted to focus on the effect of donor-recipient relatedness. Studies were also excluded if the genes needed for conjugation or replication of the plasmids were modified, or if parts of multiple natural plasmids were combined to create artificial plasmids.

The remaining studies (*n* = 345) were assessed based on their full text. Studies were excluded if the English full text was not available, if the mating experiment did not involve liquid broth matings or filter matings, if the donor species was not *E. coli*, or if no conjugation frequency was reported.

The remaining studies (*n* = 32) were included in the analysis. These involved 14 studies using liquid broth matings, 14 studies using filter matings, and 4 studies using both methods. The studies were published between 1972 and 2020 (Additional file 2).

### Data extraction

For each experiment, we recorded the donor-to-recipient ratio, the nutrient concentration, temperature, pH, and agitation of the medium, the mating time, and the pore size of the filter. For each plasmid, we recorded the Inc group. If the Inc group of the plasmid was not specified in the article, it was derived from other literature [50, 83, 84]. IncS was renamed IncH [27]. For each donor and recipient bacterium we recorded the species, their origin, growth phase, and the initial cell density. Archaic species names were replaced with the current species names as used in the Taxonomy database [85]. This database was also used to extract the taxonomic ranks genus, family, order, class, and phylum for each recipient bacterium (Additional file 3). The lowest taxonomic rank shared between the *E. coli* donor and the recipient species was used to assess their degree of taxonomic relatedness.

The conjugation frequency was expressed as the log_10_ ratio of transconjugants to donors. Data was extracted from figures using WebPlotDigitizer if needed [86]. If no transconjugants were detected, the reported detection limit was extracted. If no detection limit was reported, the detection limit was set at 1 · 10^− 6^.

### Data analysis

The data was analysed using linear mixed regression with a survival-analysis framework to account for censored data points. The log_10_-transformed conjugation frequency was used as the dependent variable. Relatedness was included as the fixed effect of interest, and study was included as a random intercept to account for the correlation between multiple measurements within studies. The conjugation frequencies that were below the detection limit were included in the statistical model as censored data points in a survival-analysis framework with the detection limits as upper bounds for the conjugation frequencies. Model selection was performed by adding variables to the model based on the lowest Akaike information criterion (AIC [87]), as long as adding a variable lowered the AIC by more than 2 points. The liquid broth matings and filter matings were analysed separately, given the fundamental differences in mating opportunities they represent. The following variables were considered for inclusion during model selection: donor and recipient origin, donor-to-recipient ratio, plasmid Inc-group, nutrient concentration, temperature, and agitation of the medium, mating time, and pore size of the filter. The growth phase and initial density of donors and recipients and the pH of the medium were not included, because in more than half of the cases they were not reported. R version 3.6.3 [88] was used for statistical analysis of the data. The survival-package version 3.1–12 was used to estimate parameters of the statistical model [89], assuming an identity link and a normal distribution of the errors.

## Supplementary information

**Additional file 1.** Reported conjugation frequencies. A graph showing the reported conjugation frequencies for liquid broth matings and filter matings.

**Additional file 2.** Number of included studies. A graph showing the number of included studies per decade for liquid broth matings and filter matings.

**Additional file 3 **Included recipient species. A table giving an overview of the included recipient species for the different levels of relatedness to *E. coli*.

## Data Availability

The datasets supporting the conclusions of this article and the R-script used for the analyses are available in the Yoda repository, 10.24416/UU01-XVLLQI.

## Abbreviations

ABR: Antibiotic resistance
*E. coli*: *Escherichia coli*
Inc: Incompatibility
