# Supplementary material for: Effect of donor-recipient relatedness on the plasmid conjugation frequency: a meta-analysis
Source: BMC Microbiol. 2020 May 26;20:135. doi: 10.1186/s12866-020-01825-4 (PMC7249681; doi:10.1186/s12866-020-01825-4)
Supplement: Supplementary file 1 — Additional file 1. Reported conjugation frequencies. A graph showing the reported conjugation frequencies for liquid broth matings and filter matings. [file 12866_2020_1825_MOESM1_ESM.docx]

**Additional file 1. Reported conjugation frequencies for liquid broth matings and filter matings.**


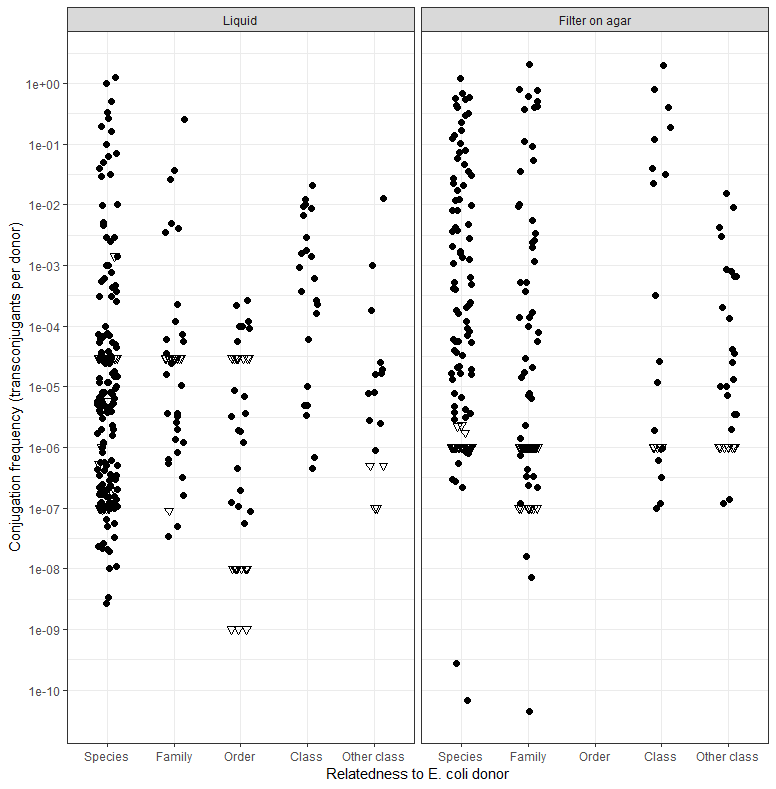


The reported conjugation frequency is plotted against the relatedness of the recipient to the *E. coli* donor. Open triangles denote the detection limit of values that were below the detection limit.
