# Supplementary material for: Effect of donor-recipient relatedness on the plasmid conjugation frequency: a meta-analysis
Source: BMC Microbiol. 2020 May 26;20:135. doi: 10.1186/s12866-020-01825-4 (PMC7249681; doi:10.1186/s12866-020-01825-4)
Supplement: Supplementary file 2 — Additional file 2. Number of included studies. A graph showing the number of included studies per decade for liquid broth matings and filter matings. [file 12866_2020_1825_MOESM2_ESM.docx]

**Additional file 2. Number of included studies per decade for liquid broth matings and filter matings.**

Articles which used both liquid matings and filter matings are only counted once in the ‘Total’ group.
