# Supplementary material for: Effect of donor-recipient relatedness on the plasmid conjugation frequency: a meta-analysis
Source: BMC Microbiol. 2020 May 26;20:135. doi: 10.1186/s12866-020-01825-4 (PMC7249681; doi:10.1186/s12866-020-01825-4)
Supplement: Supplementary file 3 — Additional file 3 Included recipient species. A table giving an overview of the included recipient species for the different levels of relatedness to E. coli. [file 12866_2020_1825_MOESM3_ESM.docx]

**Additional file 3. Included recipient species for the different levels of relatedness to *E. coli*.**

| Gram | Phylum | Class | Order | Family | Species | Number of data points | Relatedness to *E. coli* |
| --- | --- | --- | --- | --- | --- | --- | --- |
| G- | | | | | |  |  |
|  | Proteobacteria | | | | |  |  |
|  |  | Gammaproteobacteria | | | |  |  |
|  |  |  | Enterobacterales | | |  |  |
|  |  |  |  | Enterobacteriaceae | |  |  |
|  |  |  |  |  | Escherichia coli | Liquid: 184  Filter: 130 | Same species |
|  |  |  |  |  | Citrobacter freundii | Filter: 5 | Same family |
|  |  |  |  |  | Enterobacter cloacae | Liquid: 14 |  |
|  |  |  |  |  | Klebsiella oxytoca | Filter: 1 |  |
|  |  |  |  |  | Klebsiella pneumoniae | Liquid: 8  Filter: 5 |  |
|  |  |  |  |  | Lelliottia nimipressuralis | Liquid: 1 |  |
|  |  |  |  |  | Salmonella enterica | Liquid: 24  Filter: 71 |  |
|  |  |  |  |  | Shigella flexneri | Filter: 5 |  |
|  |  |  |  |  | Shigella sonnei | Filter: 1 |  |
|  |  |  |  | Erwiniaceae | |  | Same order |
|  |  |  |  |  | Erwinia amylovora | Liquid: 5 |  |
|  |  |  |  |  | Erwinia carnegieana | Liquid: 1 |  |
|  |  |  |  |  | Erwinia cytolytica | Liquid: 1 |  |
|  |  |  |  |  | Erwinia oleraceae | Liquid: 1 |  |
|  |  |  |  |  | Pantoea agglomerans | Liquid: 3 |  |
|  |  |  |  | Morganellaceae | |  |  |
|  |  |  |  |  | Proteus mirabilis | Liquid: 11 |  |
|  |  |  |  | Pectobacteriaceae | |  |  |
|  |  |  |  |  | Brenneria nigrifluens | Liquid: 1 |  |
|  |  |  |  |  | Dickeya chrysanthemi | Liquid: 2 |  |
|  |  |  |  |  | Dickeya dadantii | Liquid: 1 |  |
|  |  |  |  |  | Lonsdalea quercina | Liquid: 2 |  |
|  |  |  |  |  | Pectobacterium atrosepticum | Liquid: 1 |  |
|  |  |  |  |  | Pectobacterium carotovorum | Liquid: 2 |  |
|  |  |  |  | Yersiniaceae | |  |  |
|  |  |  |  |  | Serratia marcescens | Liquid: 12 |  |
|  |  |  | Aeromonadales | | |  | Same class |
|  |  |  |  | Aeromonadaceae | |  |  |
|  |  |  |  |  | Aeromonas sp. | Liquid: 1 |  |
|  |  |  | Pasteurellales | | |  |  |
|  |  |  |  | Pasteurellaceae | |  |  |
|  |  |  |  |  | Haemophilus influenzae | Filter: 1 |  |
|  |  |  | Pseudomonadales | | |  |  |
|  |  |  |  | Moraxellaceae | |  |  |
|  |  |  |  |  | Acinetobacter calcoaceticus | Liquid: 6 |  |
|  |  |  |  |  | Acinetobacter sp. | Liquid: 6 |  |
|  |  |  |  | Pseudomonadaceae | |  |  |
|  |  |  |  |  | Pseudomonas aeruginosa | Filter: 2 |  |
|  |  |  |  |  | Pseudomonas brassicacearum | Filter: 1 |  |
|  |  |  |  |  | Pseudomonas fluorescens | Filter: 1 |  |
|  |  |  |  |  | Pseudomonas lutea | Filter: 1 |  |
|  |  |  |  |  | Pseudomonas putida | Liquid: 10  Filter: 3 |  |
|  |  |  |  |  | Pseudomonas stutzeri | Filter: 2 |  |
|  |  |  | Xanthomonadales | | |  |  |
|  |  |  |  | Xanthomonadaceae | |  |  |
|  |  |  |  |  | Stenotrophomonas maltophilia | Filter: 2 |  |
|  |  |  |  |  | Stenotrophomonas rhizophila | Filter: 1 |  |
|  |  |  |  |  | Xanthomonas campestris | Filter: 3 |  |
|  |  |  |  |  | Xanthomonas retroflexus | Filter: 1 |  |
|  |  |  |  |  | Xanthomonas vesicatoria | Filter: 4 |  |
|  |  | Acidithiobacillia | | | |  | Other class |
|  |  |  | Acidithiobacillales | | |  |  |
|  |  |  |  | Acidithiobacillaceae | |  |  |
|  |  |  |  |  | Acidithiobacillus caldus | Filter: 2 |  |
|  |  |  |  |  | Acidithiobacillus thiooxidans | Filter: 8 |  |
|  |  | Alphaproteobacteria | | | |  |  |
|  |  |  | Rhizobiales | | |  |  |
|  |  |  |  | Brucellaceae | |  |  |
|  |  |  |  |  | Ochrobactrum rhizosphaerae | Filter: 1 |  |
|  |  |  |  |  | Agrobacterium tumefaciens | Liquid: 4 |  |
|  |  |  |  |  | Ensifer adhaerens | Filter: 1 |  |
|  |  |  | Rhodospirillales | | |  |  |
|  |  |  |  | Rhodospirillaceae | |  |  |
|  |  |  |  |  | Novispirillum itersonii | Filter: 1 |  |
|  |  |  | Sphingomonadales | | |  |  |
|  |  |  |  | Sphingomonadaceae | |  |  |
|  |  |  |  |  | Sphingomonas paucimobilis | Liquid: 3 |  |
|  |  |  |  |  | Zymomonas mobilis | Filter: 10 |  |
|  |  | Betaproteobacteria | | | |  |  |
|  |  |  | Burkholderiales | | |  |  |
|  |  |  |  | Alcaligenaceae | |  |  |
|  |  |  |  |  | Alcaligenes sp. | Liquid: 6 |  |
|  |  |  |  | Burkholderiaceae | |  |  |
|  |  |  |  |  | Burkholderia cepacia | Liquid: 1 |  |
|  |  |  |  | Oxalobacteraceae | |  |  |
|  |  |  |  |  | Janthinobacterium lividum | Filter: 1 |  |
|  |  |  | Neisseriales | | |  |  |
|  |  |  |  | Chromobacteriaceae | |  |  |
|  |  |  |  |  | Microvirgula aerodenitrificans | Filter: 1 |  |
|  | Bacteroidetes | | | | |  |  |
|  |  | Flavobacteriia | | | |  |  |
|  |  |  | Flavobacteriales | | |  |  |
|  |  |  |  | Flavobacteriaceae | |  |  |
|  |  |  |  |  | Chryseobacterium ginsengisoli | Filter: 1 |  |
|  |  |  |  |  | Chryseobacterium letacus | Filter: 1 |  |
|  |  |  |  |  | Chryseobacterium soldanellicola | Filter: 1 |  |
|  |  |  |  |  | Flavobacterium psychrolimnae | Filter: 1 |  |
|  |  |  |  |  | Flavobacterium sp. | Liquid: 1 |  |
| G+ | | | | | |  |  |
|  | Actinobacteria | | | | |  |  |
|  |  | Actinobacteria | | | |  |  |
|  |  |  | Corynebacteriales | | |  |  |
|  |  |  |  | Nocardiaceae | |  |  |
|  |  |  |  |  | Rhodococcus sp. | Liquid: 1 |  |
|  |  |  | Micrococcales | | |  |  |
|  |  |  |  | Microbacteriaceae | |  |  |
|  |  |  |  |  | Microbacterium oxydans | Filter: 1 |  |
